# Two-dimensional and tubular structures of misfit compounds: Structural and electronic properties

**DOI:** 10.3762/bjnano.5.226

**Published:** 2014-11-19

**Authors:** Tommy Lorenz, Jan-Ole Joswig, Gotthard Seifert

**Affiliations:** 1 Theoretische Chemie, Technische Universität Dresden, 01069 Dresden, Germany; 2 Helmholtz-Zentrum Dresden-Rossendorf e.V., Institute of Ion Beam Physics and Materials Research, P.O. Box 51 01 19, 01314 Dresden, Germany

**Keywords:** 2D layered materials, misfit layer compounds

## Abstract

Misfit layer compounds are structures that consist of two sublattices differing in at least one of their lattice constants. The two different layers are stacked either an alternating or in a more complex series resulting in mono- or multi-layer misfit compounds. To date, planar and bent misfit structures, such as tubes, scrolls or nanoparticles, have been synthesized and interesting magnetic and physical properties have been observed as a result of their special structures. Based on these observations, we present an overview of such misfit systems and summarize and discuss their electronic structure as well as the interlayer bonding behaviour, which is not completely understood yet. Furthermore, a more detailed insight into the SnS–SnS_2_ system is given, which was the first tubular misfit compound that has been synthesized and extensively investigated.

## Review

### Introduction

**Figure 1:**
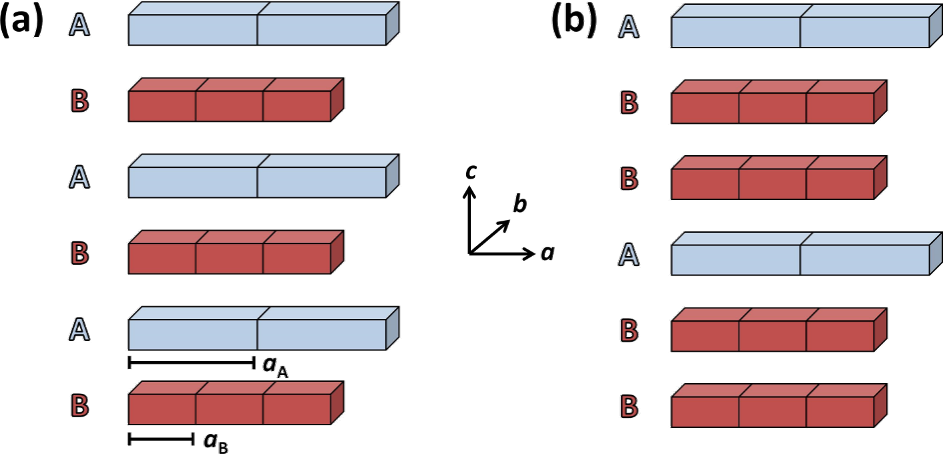
Schematic representation of two stacks of misfit compounds: (a) a misfit compound of type AB and (b) a misfit compound of type ABB.

Sheets of different two-dimensional, layered materials can assemble to form composite materials. As the compounds usually exhibit their own symmetry and space groups, their unit cells differ in most cases in either one, two, or all three directions. The combination of any two or more layers of these sheets results in a so-called misfit layer compound (MLC) [1–2], in which the difference in the compound layers leads to different effects. Figure 1a demonstrates the misfit-compound concept schematically: two different layered, 2D materials are stacked alternately in different sequences. These materials exhibit the stoichiometries MX and TMX_2_, and their total chemical formula is (MX)_1+_*_x_*(TMX_2_)*_m_* (with M = Sn, Pb, Bi, Sb, rare earths; TM = Ti, V, Cr, Nb, Ta; X = S, Se, 0.08 < *x* < 0.28, *m* = 1–3) [3]. The unit cell parameters are dissimilar at least in one direction (crystallographic direction *a* in Figure 1). The review by Meerschaut [4] considered misfit compounds with different unit cell lengths in one direction (*a*) only. Additionally, compounds that are different in both in-plane directions (*a* and *b*) are also known. For example, (SnS)_1.32_SnS_2,_ (PbSe)_0.99_WSe_2_, (PbSe)_1.00_MoSe_2_, and (SnSe)_1.03_MoSe_2_ [5–8], and misfit layer compounds consisting of other elements such as tellurium [9] or lanthanides [10] have been synthesized. Although some misfit compounds occur naturally [1,11], the recent developments in the synthesis, exfoliation, and handling of layered, two-dimensional (2D) materials gave access to the preparation of numerous misfit compounds.

To date, three comprehensive reviews have dealt with planar [3–4] and tubular [6] misfit compounds, which were as well the foundation for the present overview. In the following, planar and tubular misfit structures are discerned and their individual structural and electronic properties are discussed. Note that the following sections are sorted with respect to the reviewed properties. A section containing special examples will be followed by a summary.

### Structures

**Figure 2:**
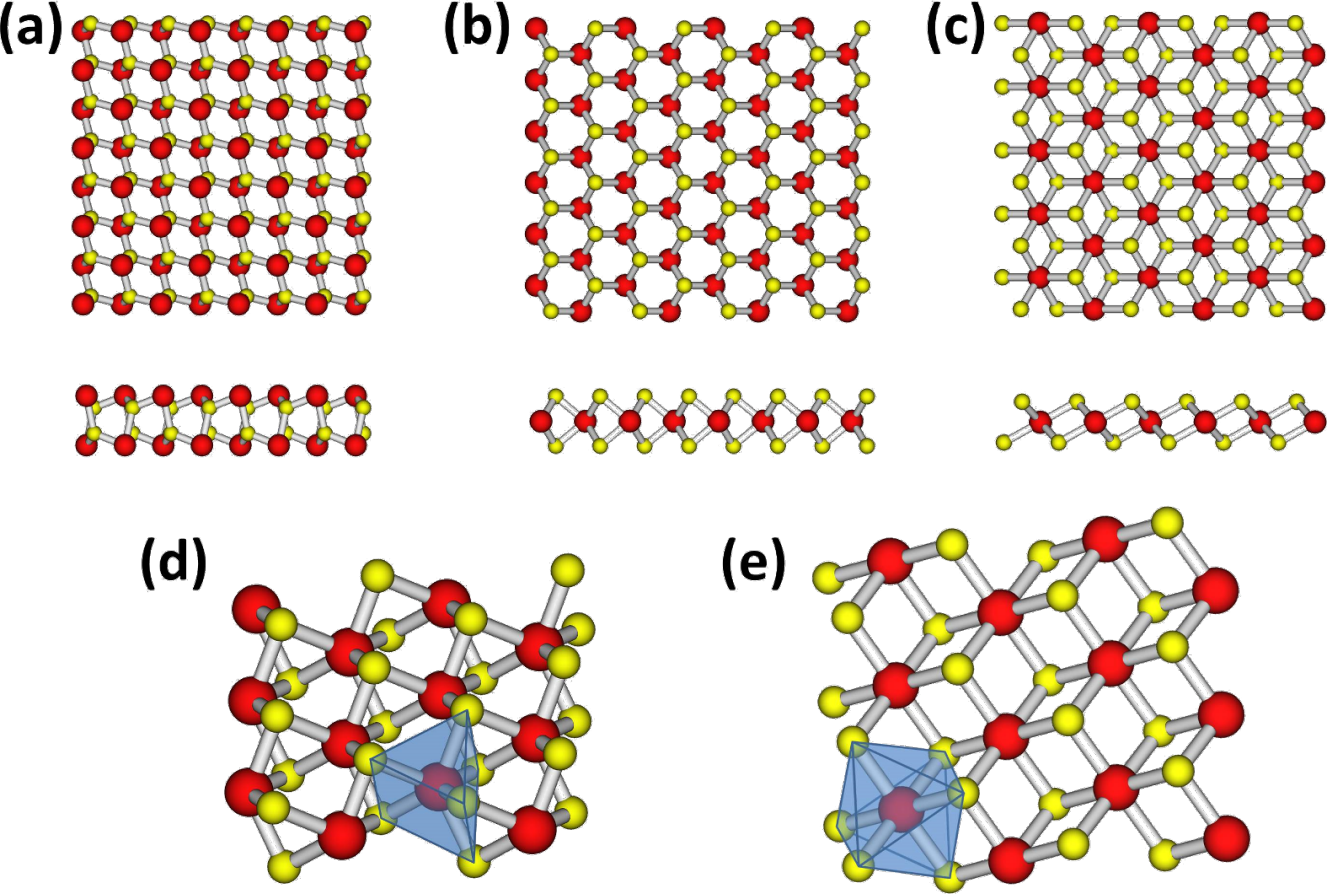
Structures of a monolayer of (a) MX and (b–e) TMX_2_. The TMX_2_ layer occurs in two configurations with the metal atoms coordinated either (b)/(d), trigonal prismatically, or (c)/(e), octahedrally. The metal and chalcogenide atoms are shown as red and yellow spheres, respectively.

Layered chalcogenides most commonly form misfit compounds [3] with stoichiometries MX/TMX_2_ (chemical formula (MX)_1+_*_y_*(TMX_2_)*_m_* with M = Sn, Pb, Bi, Sb, rare earths; TM = Ti, V, Cr, Nb, Ta; X = S, Se , 0.08 < *y* < 0.28, *m* = 1–3). The lengths of the lattice vectors 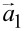 and 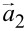 (the indices label the two components of the misfit compound) in the mismatched direction determine the ratio of MX to (TMX_2_)*_m_*. This is expressed in the sum formula by the variable *y* and can be derived as *y* = (4/2)·(*a*_2_/*a*_1_) − 1. The individual layer types, that is, MX and TMX_2_, have different stoichiometries, and thus, differing structures. Whereas MX layers show a distorted rock salt structure (Figure 2a), the TMX_2_ layers have a sandwich structure, where a sheet of (transition) metal atoms is sandwiched by two layers of chalcogenide atoms (Figure 2b,c). The transition metal atoms can assemble in a trigonal prismatic or octahedral coordination in the TMX_2_ layer (Figure 2d,e). The TMX_2_ labeling is according to the polytypes of their lamellar compounds, that is, their stacking order (number of layers in the unit cell) and symmetry (T, H, R for trigonal, hexagonal, rhombohedral symmetry, respectively) generate the labels.

As can be seen in Figure 1, different sequences of stacking are possible: Either an alternating A–B–A–B–… stacking (Figure 1a) or bi- (*m* = 2) and trilayer (*m* = 3) systems are known. Here, the parameter *m* indicates the number of successive layers of the same type. In the latter two cases, two or three TMX_2_ layers follow directly and are embedded into two MX layers as shown in Figure 1b. In these cases, the TMX_2_ layers are held together by van der Waals forces, whereas the interaction between MX and TMX_2_ layers is based on van der Waals interaction and a charge transfer (CT) from MX to TMX_2_ [12]. Thus, misfit compounds do not only differ by stoichiometry, difference in structure, and individual coordination in the TMX_2_ layer, but also by the ratio between the two subsystems, MX and TMX_2_.

#### Planar structures

We will now focus on a discussion of crystallographic data obtained from different planar misfit compounds. In most cases, the angle between the in-plane lattice vectors 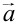 and 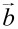 (as defined in Figure 1) is 90°, so that the 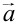 and 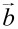 vectors in both interacting compounds are parallel. If the 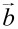 vectors have the same length, 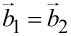 results. An exception is reported by Ren et al. [13] for (SbS)_1.15_TiS_2_ with angles of γ_1_ = 84.06° and γ_2_ = 90.01°. Generally, the differences occur regularly in both directions as a result of right-angled in-plane lattice vectors.

Most of the systems with NbX_2_ or TaX_2_ sublayers in a trigonal prismatic coordination have α angles of nearly 90°. There are, however, misfit compounds with different 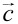 vectors and α or β angles deviating from 90°, for example, (YS)_1.23_NbS_2_ [14] and (HoS)_1.23_NbS_2_ [15] with trigonal prismatic TMX_2_ layers, monoclinic (PbS)_1.18_TiS_2_ [16] and (PbS)_1.12_VS_2_ [17], or triclinic (LaS)_1.20_CrS_2_ [18] and (SnS)_1.20_TiS_2_ [19], all four with octahedral coordination in the TMX_2_ layer. The unit cell parameters of some misfit compounds are given in Table 1.

**Table 1:** Cell parameters of different misfit layer compounds. This table has been taken from Rouxel et al. [3].

Compound	*a*_1_ [Å]	*a*_2_ [Å]	*y* = 2·(*a*_2_/*a*_1_) – 1	*b*_1_ = *b*_2_ [Å]	*c*_1_ = *c*_2_ [Å]	α [deg.]	Ref.

(SnS)_1.17_NbS_2_	5.673	3.321	0.1708	5.751	11.761	90	[20–21]
(LaS)_1.14_NbS_2_	5.828	3.310	0.1359	5.797	*c*_1_ = 11.52^a^ *c*_2_ = 23.94	90	[22–23]
(PbS)_1.18_TiS_2_	5.800	3.409	0.1755	5.881	11.76	95.28	[16]

^a^*c*_2_ = 2*c*_1_.

As mentioned above, different types of stacking are possible for these composite structures. They may differ, for example, in the number of sub-layers, their symmetry and orientation. The most common structures are 2*H* and 3*R*. Due to the weak interlayer forces, layer exfoliation is easily possible [24].

**Figure 3:**
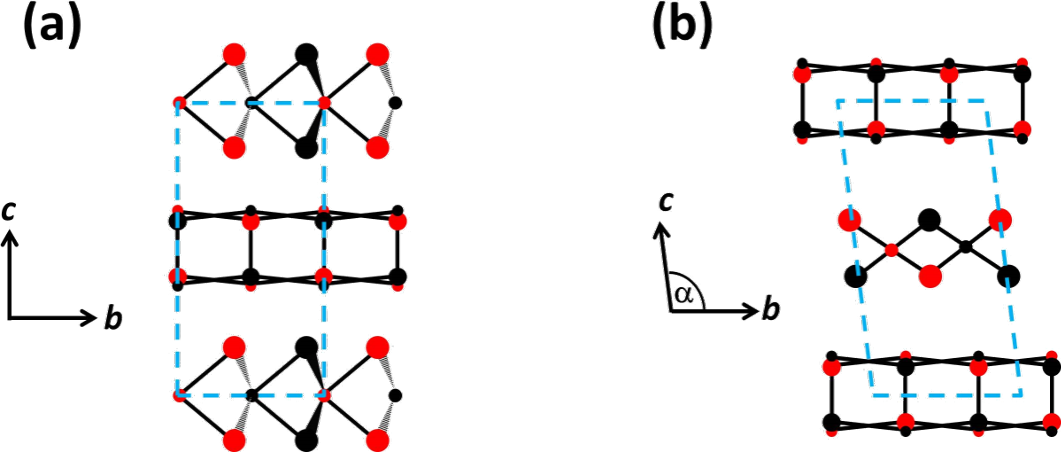
Side view of two unit cells of misfit layer compounds with the TMX_2_ component either in trigonal prismatic (a) or octahedral coordination (b). The unit cells are denoted by the blue boxes. Adapted with permission from [3]. Copyright 1995 Elsevier.

As a consequence of their structure, interaction between the M atoms of the MX layer and the X atoms of the TMX_2_ layer exists in all misfit compounds. In each unit cell this interaction occurs twice: at the top and the bottom sides of the layers. This can be seen in Figure 3. Additionally, this figure shows that the symmetry of the misfit compound’s unit cell is determined by the sublayers and their symmetries. The metal atoms of the TMX_2_ sublayer can be coordinated trigonal prismatically by the chalcogenide atoms (Figure 3a), so that the symmetry of the whole cell is orthorhombic as, for example, in (SnS)_1.17_NbS_2_. On the other hand, for a monoclinic misfit compound such as (PbS)_1.18_TiS_2_, the TMX_2_ sublayers have a (distorted) octahedral symmetry (Figure 3b).

**Figure 4:**
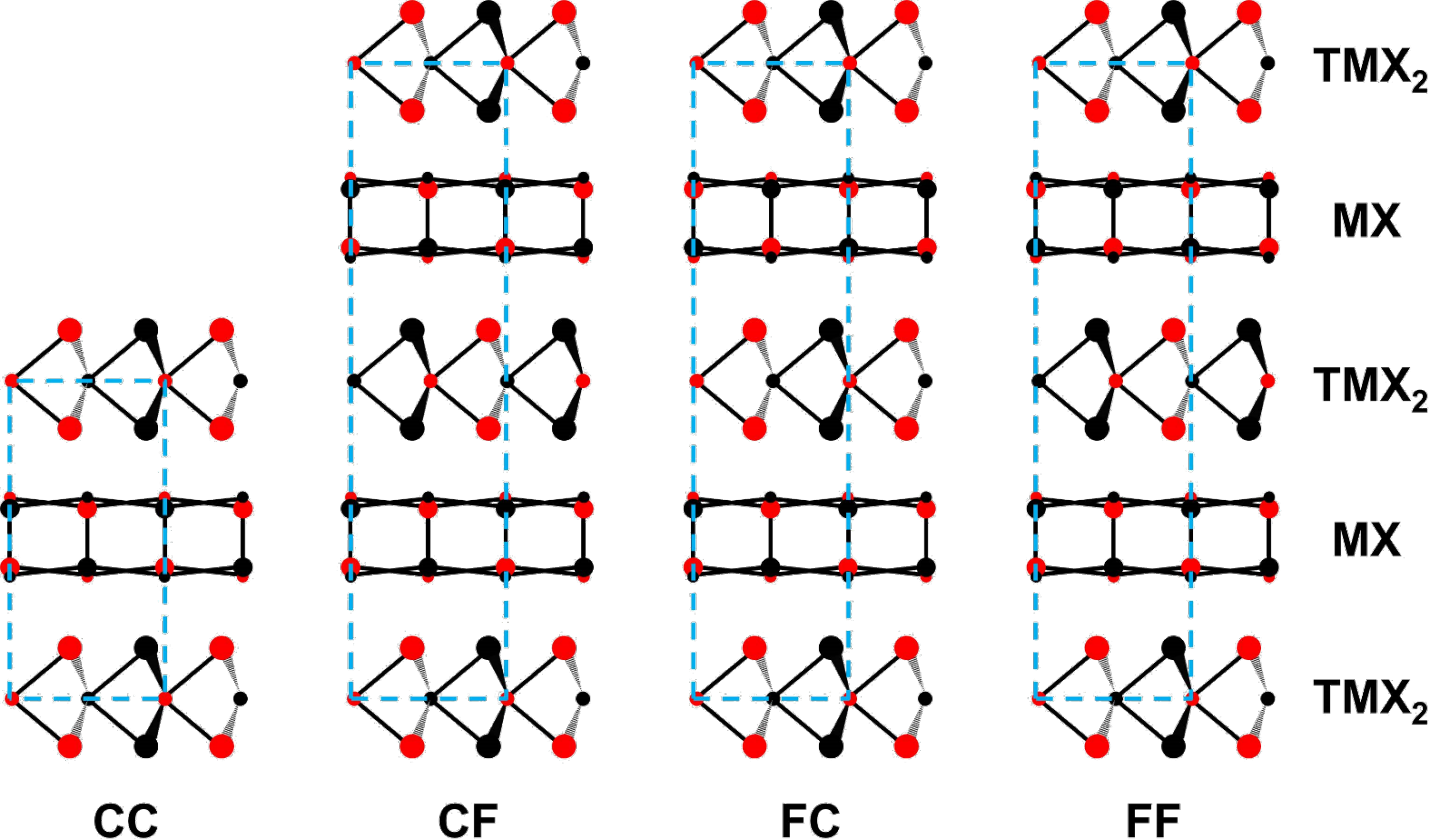
Different possible stacking types of misfit compounds projected along the [100] direction. The blue boxes denote the unit cell and the colored circles represent the metal (small circles) and chalcogenide atoms (large circles), where the different colors represent the different positions in space: black atoms above the paper-plane and red ones beneath it. Adapted with permission from [3]. Copyright 1995 Elsevier.

In cases where the misfit occurs in only one direction, for example, in *b*, the number of stacking possibilities grows, since the individual layers may be centered differently with respect to the *b* direction (see Figure 4). Each subsystem has either a so-called C-centered or an F-centered lattice. In both cases, the *c* axes match. In the F-centered structure, the length of the *c* axis in the unit cell is doubled due to an additional shift of 1/2*c*. Accordingly, four different possibilities of stacking are possible: CC, CF, FC, and FF (see Figure 4). In the CC system, the two *c* axes of the subsystems completely match (in direction and length) which is the case, for example, in the compound (SnS)_1.17_NbS_2_. In the FC system, *c*_1_ = 2∙*c*_2_ as it occurs in, for example, (YS)_1.23_NbS_2_, whereas *c*_2_ = 2∙*c*_1_ is seen in CF systems such as (LaS)_1.14_NbS_2_. In misfit compounds of the FF type, both 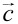
vectors have the same direction and norm, but twice the length as the CC type. The structure of (PbS)_1.13_TaS_2_ is an example of the latter type.

#### Non-planar structures

In addition to the previously discussed planar misfit structures, bent systems are common as well [5–6,25–29]. Such tubular or cylindrical misfit layer compounds have been mentioned for the first time in relation to the mineral cylindrite [1,30]. Generally, it is assumed that a misfit in *a* and *b* directions leads to the formation of tubular structures by reason of energy minimizing [6]. The advantage of these structures is the absence of any dangling bonds, which leads to more energetically stable systems compared to the finite planar ones (stripes, flakes, platelets) [31–32]. Hence, the act of bending or rolling can be seen as a relaxation process, although the relative thickness of the tube walls and the rigidity of the interatomic bonds were seen as a steric hindrance. The rolling process has been discussed in relation to the misfit compounds (PbS)_1.14_(NbS_2_)_2_ [29], (BiS)_1.17_(NbS_2_)*_n_* [33] with *n* = 1–4 or (SnS)_1.32_SnS_2_ [25]. Just as for planar misfit compounds, different stacking types are possible in the bent misfit systems as well regarding the number of sublayers, or rather, the stacking order (as shown in Figure 1), and also regarding the possibility of different structures and orientations of the sublayers, as shown in Figure 3 and Figure 4. For example, several different stacking configurations have been observed for (SnS)_1.32_(SnS_2_)*_n_*, such as ABAB, ABBABB, ABABBABABB [6,26]. As in planar misfit systems, the composite has a global super-symmetry that may differ from that of the two subsystems. Due to two different lattice vectors (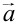 and 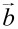), multiple in-plane orientations of the sublayers are possible, which leads to different rolling vectors and therefore, to a manifold of chiral nanotubes. However, aside from tubular misfit layer compounds, nanoscrolls and nanoparticles containing sublayers with different lattices were produced [5–6,25–26], which can have the same structural parameters (stacking order, number and orientation of sublayers, etc.) as tubes and planar misfit compounds.

### Electronic structure and interlayer bonding

Meerschaut [4] suggests that the properties of misfit layer compounds might be attributed to the properties of the individual compounds or subsystems. For example, the van der Waals gap in TMX_2_ systems can be chemically intercalated [34]. A misfit compound can, thus, be viewed as a TMX_2_ system intercalated by another layered MX system. As a consequence, the electronic properties of some misfit compounds have been successfully described by a rigid-band formalism. In this description, the electronic bands are taken as immutable characteristics and only the filling is changed depending on the intercalated species.

In addition to theoretical considerations, the electronic structure is discernible by spectroscopy as Ohno [12,35] presented in 1991. By performing X-ray photoelectron and absorption spectroscopy (XPS, XAS) and reflection electron energy loss spectroscopy (REELS), it was revealed that the electronic structure indeed can be well-described by a superposition of bands of individual single layers. From these results it was concluded that a charge transfer from MX to TMX_2_ takes place, which is independent from the nature of the metal M (either M^2+^: Sn, Pb, or M^3+^: La, Ce, Sm). In that case, MX can be viewed as a donor and TMX_2_ as an acceptor of the transferred electron density. This claim was based on studies of valence band XPS and XAS spectra of the misfit compounds, compared to those of the individual layers, and a spectrum of iron intercalated titanium disulfide (Fe_1/3_TiS_2_), which can be interpreted as a true intercalated system. Figure 5 in [35] shows this XAS spectra of the *K* absorption edge of sulfur for the systems TiS_2_, PbS, PbTiS_3_ (=MLC (PbS)_1.18_TiS_2_), and Fe_1/3_TiS_2_. The shapes of the PbTiS_3_ and Fe_1/3_TiS_2_ spectra are quite similar. From this, charge transfer was concluded, which should result from the filling of the *t*_2_*_g_* energy levels coming from the titanium d states.

**Figure 5:**
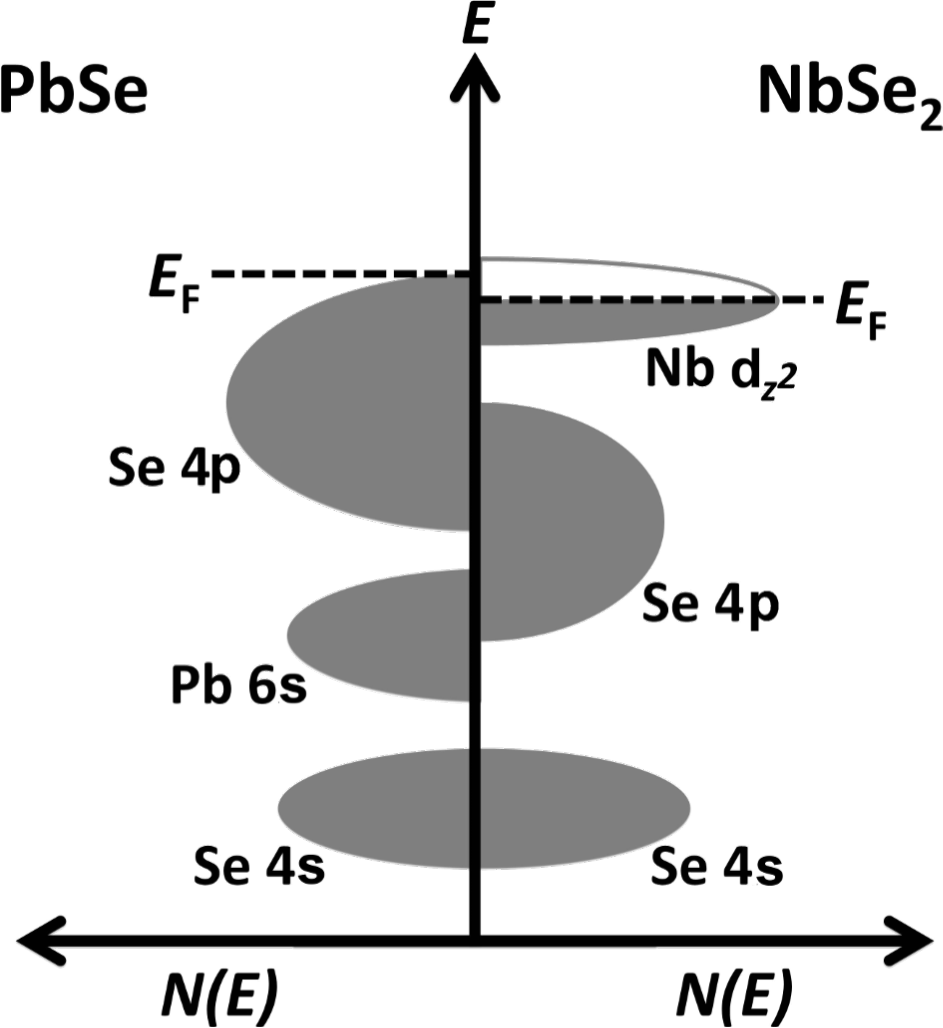
Schematic representation of the density of states of a PbSe layer (left) and a NbSe_2_ layer (right). The electron transfer takes place from the PbSe valance band to the conduction band of the NbSe_2_ layer. Adapted with permission from [36]. Copyright 2014 American Chemical Society.

Further studies used a comparable argument for the claim of charge transfer in other misfit layer compounds. One example is the electron transfer from PbSe to NbSe_2_ in [(PbSe)_1.14_]*_m_*(NbS_2_)_1_ with *m* = 1–6 [36]. The electronic structure has been predicted by density functional theory calculations using the generalized gradient approximation. From the overlap of the empty bands in NbS_2_ with the filled valence band of PbSe, the authors concluded that “only a small fraction of an electron’s charge is transferred per atom” [36]. In Figure 5 the density of states of the electronic bands of PbSe and NbSe_2_ near the Fermi level are displayed schematically. The overlap of the Se(PbSe) 4p states with the Nb d_z²_ states can be seen clearly, which is used as an argument for potential charge transfer. Additionally, the dependence on the number of PbSe sublayers in one unit cell of the misfit compound has been investigated (varying *m* in the sum formula) with the result that the interlayer charge transfer increases with increasing *m*.

Another theoretical work [37] analysed the band structure of (SnS)_1.17_NbS_2_ by using a ratio of 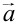 vectors of 5:3 resulting in a (SnS)_1.20_NbS_2_ stoichiometry. The authors concluded a charge transfer of 0.4 electrons per niobium atom from their calculations. Furthermore, they showed that the Sn 5s orbitals mix strongly with the S 3p orbitals from NbS_2_.

Moreover, vibrational spectroscopy has been used to investigate a possible interlayer charge transfer. The Raman spectra of (SnS)_1.17_NbS_2_ and (PbS)_1.18_TiS_2_ (powdered samples and single crystals) as well as single crystals of (PbS)_1.14_NbS_2_ were compared to those of the pure substances and showed a superposition of the interlayer vibrations of the individual layers. Shifts in the NbS_2_ modes relative to those in 2H-NbS_2_ were interpreted to be a result of charge transfer. These spectra are published in [38–39].

In contrast to the previously shown examples, Ettema and Haas [40] performed XPS measurements for the core states of misfit layer compounds. This work can be seen as an extension of Ohno’s work [12,35], which was limited to the valence bands and has been discussed above. They showed that tin and lead are divalent in compounds of the structure (MX)_1+_*_y_*TMX_2_ due to the fact that the core-level binding energies of these elements do not differ in either the misfit compounds or the isolates systems (SnS, PbS). If a (small) charge transfer took place, the core-level energies of the TM atoms in the TMS_2_ sublayer would have stayed nearly constant. Experimentally, this shift in binding energy was not observed for the 2s, 2p, 3s, 3p levels of Ti, the 3s, 3p, 3d, 4s, 4p levels of Nb and the 4p, 4d, 5p, and 4f levels of Ta. From this fact, the authors concluded that there is no significant interlayer charge transfer and the stability of the misfit layer compounds results from covalent bonds between the several sublayers.

Moëlo et al. [41] investigated different misfit systems consisting of Sn and Pb (single-, double- and triple-layer, i.e., *m* = 1–3) using electron microprobe analysis. Since the M and TM atoms are in oxidation states +2 and +4, respectively, each sublayer should be uncharged and electronically balanced. Hall and Seebeck measurements performed by Auriel et al. [42] showed a highly anisotropic metallic behaviour of misfit layer compounds; some of them even showed a transition to superconductivity at temperatures less than 6 K. A small intrinsic charge transfer could explain such physical properties. However, it is quite improbable that (especially) lead has oxidation states higher than +2 in a Pb–S system. Thus, a charge transfer from Pb (MX) to TMX_2_ is unlikely. The concept of cationic coupling assumes an M (e.g., Pb or Sn) deficiency in the MX layer and therefore a TM (e.g., Nb) excess. In fact, some of the M^2+^ cations in the MX sublayer are substituted by TM^3+^ ions. The excess of positive charge must then be adjusted by reduction of the same amount of TM^4+^ ions in the TMX_2_ layer. Thus, the MX sublayer is positively charged and, as a consequence, the TMX_2_ layer becomes negative, so that a charge transfer is introduced. The resulting strong, attractive, electrostatic interaction between the two sublayers is the origin of the stability of the misfit compounds within the concept of cationic coupling.

To conclude this section, we will briefly mention the so-called graphite intercalation compounds (GICs) [43] that consist of several graphene layers, which are intercalated by different atoms or fragments, for example, alkali and alkaline earth derivatives or metal halide derivatives. Those components are held together by charge transfer and not every van der Waals gap is intercalated by guest atoms. The systems are called “stage 1 compounds” in the case of an alternating arrangement, and “stage 2 compounds”, if every second graphene layer is intercalated, and so on. In contrast to misfit structures, which were the focus of this review, the host molecules and atoms in GICs are intercalated and do not have the same or comparable lattice structure as they have in their bulk phases. For this reason we will not go beyond mentioning GICs here and refer the interested reader to the respective literature.

### Examples

#### SnS–SnS_2_ layers and nanotubes

**Figure 6:**
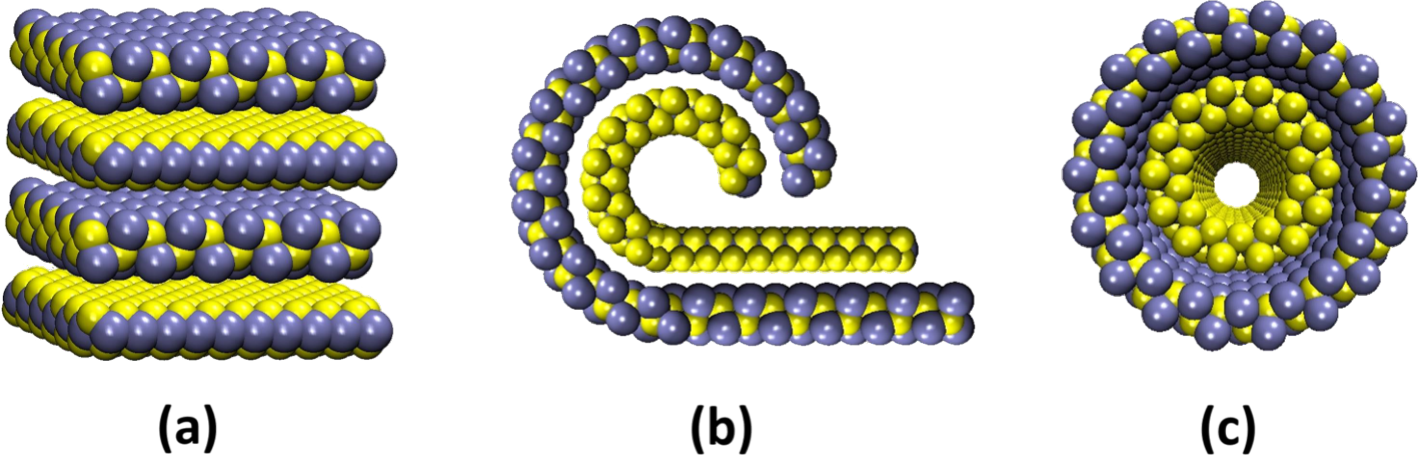
SnS–SnS_2_ misfit compounds forming nanoscrolls and nanotubes. Yellow spheres represent sulfur atoms and blue spheres tin atoms.

In 2003, inorganic fullerene-like nanoparticles and small nanotubes were synthesized [5]. These structures have spherical or polyhedral shapes and consist of orthorhombic SnS and hexagonal SnS_2_ lattices. The two sublattices have the same structure and orientation as the “classical” layered misfit compounds discussed above. Meanwhile, SnS–SnS_2_ nanostructures have been further produced, such as nanotubes and nanoscrolls (see Figure 6). These structures have been extensively experimentally and theoretically investigated to date [6,25–26,44]. Selected area electron diffraction (SAED) measurements showed that the interlayer distance between the SnS and SnS_2_ substructures is nearly unchanged relative to the bulk structures (error 3%) [26]. For this reason, it is estimated that the sublattice structures in the SnS–SnS_2_ system are almost the same as in the pristine bulk material. As a consequence, both in-plane directions are different from one another. The resulting SnS–SnS_2_ nanotubes result in the stoichiometries (SnS)_1.32_SnS_2_, (SnS)_1.32_(SnS_2_)_2_ and [(SnS)_1.32_]_2_(SnS_2_)_3_. SAED patterns further show that the SnS_2_ subsystems in these misfit tubes have two different rolling directions. This is equivalent to two different SnS_2_ chiralities (zig-zag, armchair tubes) in the misfit tube.

A recent theoretical study performed with density functional-based tight-binding calculations investigated a possible charge transfer between the SnS and SnS_2_ layers. By comparing the density of states (DOS) for a hypothetical SnS–SnS_2_ misfit system with the sum of the DOSs of the two isolated monolayers, the authors showed that additional states arise in the gap region hinting at an electronic interaction between the layers. Furthermore, the atomic charges in the combined system obtained by Mulliken population analysis surprisingly showed that the SnS_2_ layer was slightly positively charged, whereas the SnS layer was negatively charged. Although the received charge transfer is quite small (approx. 0.1 electrons per SnS_2_ unit) the direction of the electron transfer was perplexing, especially because this direction is unexpected because the Sn^4+^ system transfers electron density to the Sn^2+^ system. The explanation might be that the charge transfer does not take place from metal to metal (Sn^2+^ → Sn^4+^), because Sn is not a transition metal, in contrast to the TMX_2_ systems discussed above. Thus, the sulfur atoms of the SnS_2_ layer could act as donors and Sn^2+^ atoms in the SnS layer as acceptors. Generally, Sn^2+^ can act as an acceptor if a stronger reducer is present. By comparing the standard potentials of the S^2−^/S and Sn/Sn^2+^ systems, it appears that this explanation is possible and is supported by the fact that in the SnS_2_–SnS bilayer, only the SnS_2_-sulfur atoms facing the SnS layer become slightly positive (see Figure 5 in [44]).

#### SiO_x_-coated carbon nanotubes

**Figure 7:**
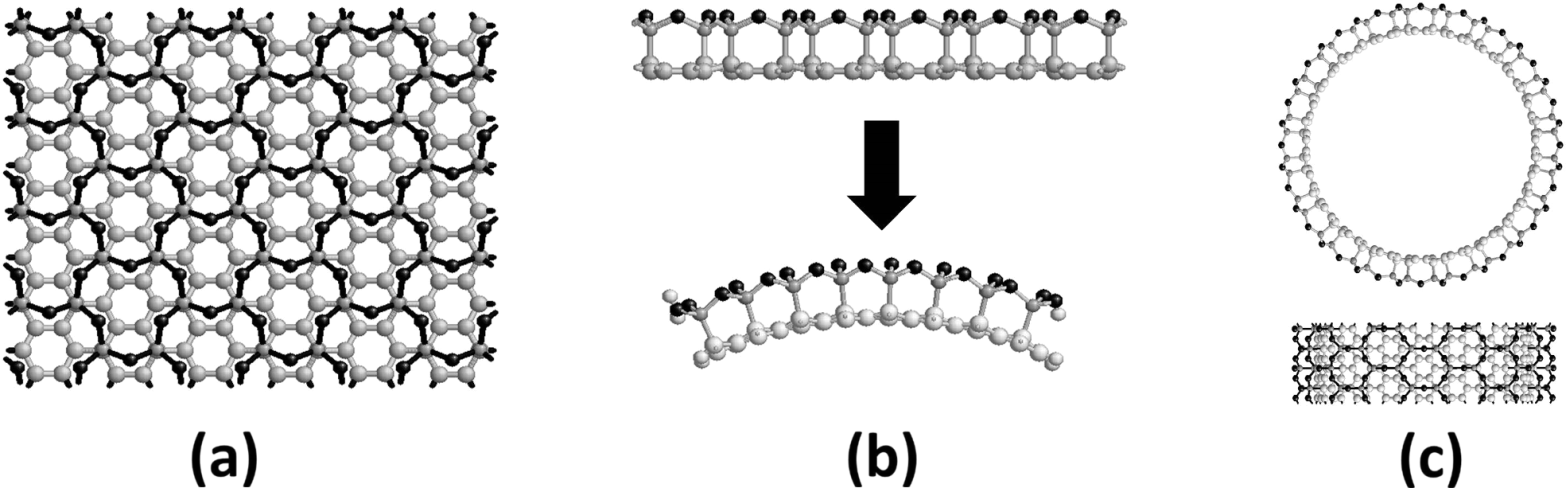
Superposition of the hexagonal carbon lattice and the six-membered SiO_4_ rings. (a) Top view, (b) side view with the depiction of the spontaneous bending of the layers and the C–Si bonds and (c) a carbon nanotube is surrounded by a cylindrical SiO_x_ (x = 5/2) layer. Carbon, oxygen, and silicon atoms are colored in light gray, dark gray, and black, respectively.

In 2002, carbon–SiO_x_-based nanocomposites were synthesized [27–28]. Although these structures differ significantly from the discussed misfit compounds, the two host lattices adjust and the mechanical strain is reduced by a spontaneous bending. Figure 7a shows the two planar sublattices in which the hexagonal carbon structure (graphene) and the six-membered SiO_4_ rings align their lattice constants. To minimize the energy, the C–Si-bonded system bends spontaneously (Figure 7b). This relaxation process leads to the formation of tubular structures and Si–O–Si bond angles of 140°. In this system, a real covalent bond between the two subsystems can be observed, which partly changes the hybridization of the carbon atoms from sp^2^ to sp^3^.

## Conclusion

In this review, the structures of misfit layer compounds were discussed. As was illustrated, MLCs consist of two different sublattices with at least one lattice vector of different length in the subsystems. By aligning their lattices, the layers with stoichiometries MX and TMX_2_ can be stacked alternating or in more complex sequences connecting to a misfit compound. They can adopt planar or bent structures, and the resulting super-symmetry usually differs from the symmetry of the pristine systems. To date, layers, tubes, nanoparticles, and nanoscrolls formed by misfit layer compounds are known and have been synthesized. In the non-alternating A–B–B–… stacking, the successive layers of the same type are bonded only by van der Waals interaction. This is one reason why it is possible to intercalate metal atoms within this van der Waals gap. Due to the fact that layered materials show exceptional lubricating properties originating in strong intra-layer, but weak inter-layer interactions, lubricating properties can be expected for misfit layer compounds as well.

For the purpose of structure determination, many measurements and experiments have been performed for misfit layer compounds. Furthermore, the determination of their electronic structure has been implemented considerably. From this data, estimations of the bonding behaviour between the sublayers were made. On the one hand, a charge transfer from the MX to the TMX_2_ layer is mentioned, on the other hand the concept of cation coupling is discussed, which assumes the substitution of M^2+^ by TM^3+^ cations in the MX layer. This might introduce a charge transfer through the necessity of electroneutrality for the whole system. The presented concepts may explain the stability of the misfit layer compounds and their properties, but further studies are necessary for a comprehensive description of these interesting systems.
